# Machine Learning Assessment of Background Parenchymal Enhancement in Breast Cancer and Clinical Applications: A Literature Review

**DOI:** 10.3390/cancers16213681

**Published:** 2024-10-31

**Authors:** Katie S. Duong, Rhianna Rubner, Adam Siegel, Richard Adam, Richard Ha, Takouhie Maldjian

**Affiliations:** 1 Department of Radiology, Montefiore Medical Center, Albert Einstein College of Medicine, Bronx, NY 10467, USA; 2007katieduong@gmail.com (K.S.D.); rrubner@montefiore.org (R.R.); adambsiegel@gmail.com (A.S.); rha@montefiore.org (R.H.); 2 New York Medical College, 40 Sunshine Cottage Road, Valhalla, NY 10595, USA; radam2@student.touro.edu

**Keywords:** BPE, breast cancer, magnetic resonance imaging, MRI, contrast-enhanced MRI, artificial intelligence, deep learning, convolutional neural network

## Abstract

Background Parenchymal Enhancement (BPE) on breast MRI holds promise as an imaging biomarker for breast cancer risk and prognosis. The ability to identify those at greatest risk can inform clinical decisions, promoting early diagnosis and potentially guiding strategies for prevention such as risk-reduction interventions with the use of selective estrogen receptor modulators and aromatase inhibitors. Currently, the standard method of assessing BPE is based on the Breast Imaging-Reporting and Data System (BI-RADS), which involves a radiologist’s qualitative categorization of BPE as minimal, mild, moderate, or marked on contrast-enhanced MRI. This approach can be subjective and prone to inter/intra-observer variability, and compromises accuracy and reproducibility. In addition, this approach limits qualitative assessment to 4 categories. More recently developed methods using machine learning/artificial intelligence (ML/AI) techniques have the potential to quantify BPE more accurately and objectively. This paper will review the current machine learning/AI methods to determine BPE, and the clinical applications of BPE as an imaging biomarker for breast cancer risk prediction and prognosis.

## Simple Summary

Background Parenchymal Enhancement (BPE) on breast MRI holds promise as an imaging biomarker for breast cancer risk and prognosis. Machine learning/artificial intelligence (ML/AI) techniques have the potential to quantify BPE more accurately and objectively. This paper will review the current machine learning/AI methods to determine BPE, and the clinical applications of BPE as an imaging biomarker for breast cancer risk prediction and prognosis.

## 1. Background

The diversity and heterogeneity of breast cancer does not lend itself to a simplistic approach and predicting which women will develop breast cancer remains elusive. While many risk factors for breast cancer have been identified, most women who develop breast cancer have no known risk factors. Imaging may help to better elucidate breast cancer risk by shedding light on the tumor microenvironment, the final common pathway in triggering carcinogenesis. Background parenchymal enhancement (BPE) [1], defined as the extent of normal fibroglandular tissue (FGT) enhancement observed on breast contrast-enhanced MRI, has evolved from the notion that BPE might be a confounding factor that obscures detection of breast cancers to now being widely acknowledged as an imaging biomarker that could be helpful in predicting breast cancer risk, treatment outcome, and recurrence risk. Physiological variables that influence BPE include vascular density, endothelial growth factors, permeability of blood and lymphatic vessels, and hormonal levels (including menstrual status). There is growing evidence that BPE is a marker for breast cancer risk. Recent studies suggest a significant correlation between elevated BPE levels and an augmented risk of developing breast cancer and increased likelihood of breast cancer occurrence. 

King et al. demonstrated that increased BPE on breast MRI is a significant predictor of the presence of breast cancer, and the odds ratio increased with increasing BPE [2]. Dontchos et al. showed that BPE is associated with increased risk of developing breast cancer on follow-up [3]. Moreover, basal BPE and changes in BPE have the potential to predict a patient’s response to neoadjuvant breast cancer treatment and overall treatment outcomes, providing clinicians with additional insights for tailoring treatment approaches. These findings are particularly timely given the ongoing paradigm shift in breast cancer screening and treatment strategies towards more targeted and individualized approaches.

While BPE has the potential to serve as an important independent biomarker for breast cancer risk, treatment outcomes, and recurrence risk, the challenge lies in accurately segmenting fibroglandular tissue (FGT) for quantitative BPE measurements, necessitating advanced imaging techniques and analysis methods. There is also no consensus regarding the optimal timing for dynamic contrast-enhanced MRI in BPE quantitation. Refining these methodologies becomes imperative to unlock the full potential of BPE as a clinically relevant biomarker.

The goal of this review paper is to summarize current machine learning methods to estimate BPE, and the current clinical applications of BPE as an imaging biomarker to predict breast cancer risk, treatment outcome, and recurrence risk.

## 2. Methods

**Figure 1:**
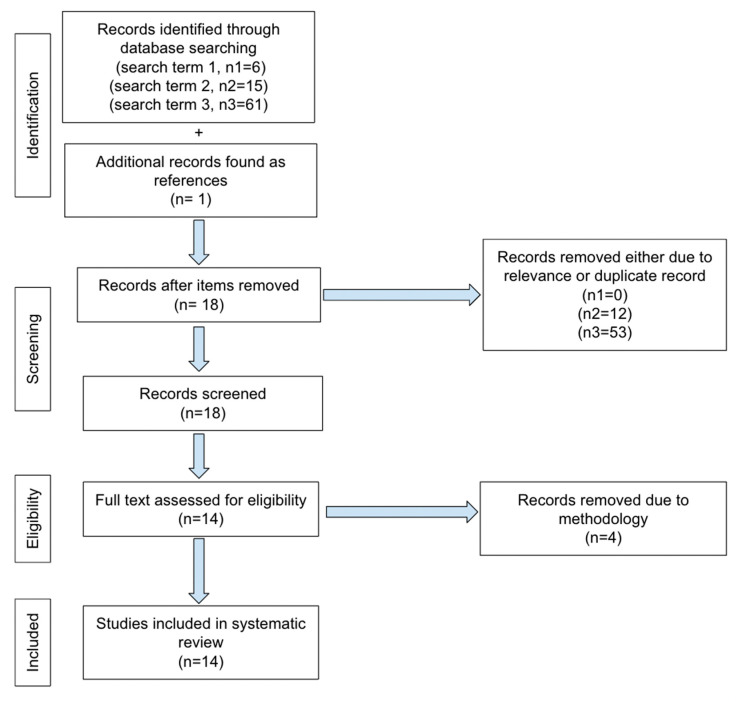
PRISMA selection flowchart.

No ethics committee approval was required for this systematic review. PRISMA guidelines for reporting were adopted in our review. The literature search was performed from March 2017 to February 2024 using the following keywords on PubMed: “MRI and BPE and machine learning” and “MRI and BPE and artificial intelligence” and “quantitative background parenchymal enhancement and MRI”. The first search terms yielded 6 total articles. One of the 6 was excluded as it only pertained to segmentation methodology. There were 5 articles that met our inclusion criteria and dates. The second search term yielded 15 total articles, of which 8 pertained to our subject, however only 2 were new and one concerned segmentation. The 3rd search term yielded 61 total articles, of which 8 were new articles. However, 2 were focused on segmentation methodology and only 6 applied. Only one additional article was found as a reference from articles obtained by our search terms. The total number of articles is 14 for our review, shown in our PRISMA selection flowchart (Figure 1). 

KSD, RR, and AS performed and verified searches and results were confirmed by TM.

### Common Machine Learning Algorithms Appeared in This Review

A CNN is a deep learning model primarily used for image-related tasks like classification, object detection, and segmentation. It consists of convolutional layers that automatically extract features from input images by applying filters. These filters detect edges, textures, and complex patterns, progressively learning more abstract representations. CNNs use pooling layers to reduce the spatial dimensions and fully connected layers for final classification. Their ability to process raw image data with minimal preprocessing makes them highly effective in computer vision tasks. VGG16 is a deep CNN architecture with 16 layers (13 convolutional and 3 fully connected). It uses small 3 × 3 convolution filters stacked sequentially, followed by max pooling layers to reduce dimensionality. VGG16 was developed for the ImageNet challenge and is known for its simplicity and high accuracy in image classification. The pre-trained VGG16 model is widely used for transfer learning in applications that require high-performance image recognition. VGG19 is an extension of VGG16 with 19 layers (16 convolutional and 3 fully connected). U-Net is a CNN designed for image segmentation, particularly in medical imaging. It features an encoder-decoder structure with symmetric skip connections. The encoder captures features, while the decoder upsamples them to generate detailed segmentation maps, making it highly effective for tasks requiring precise boundary delineation.

Fuzzy C-Means clustering allows data points to belong to multiple clusters with varying degrees of membership, making it ideal for cases with unclear cluster boundaries, such as image segmentation. It minimizes a cost function that considers both distance to cluster centers and fuzziness in assignments. K-means clustering, by contrast, assigns each data point to the nearest of a predetermined number of clusters. It iteratively updates cluster centers to minimize within-cluster variance. K-means is efficient and widely used for data segmentation but requires specifying the number of clusters in advance and assumes uniform cluster shapes.

CNNs excel at image-related tasks by automatically extracting features but require large datasets and can be computationally intensive. VGG16 and VGG19 offer high accuracy in image classification, yet their complexity leads to longer training times and potential overfitting. U-Net is effective for image segmentation, particularly in medical imaging, but needs substantial annotated data. Fuzzy C-Means clustering accommodates overlapping clusters, making it suitable for ambiguous boundaries, but it is more computationally intensive. In contrast, K-Means is efficient and simple but requires predefining the number of clusters and assumes uniform shapes, limiting its applicability in complex datasets.

## 3. Results

We reviewed 4 papers on using ML to classify BPE levels (Table 1), 2 papers on using ML to predict breast cancer recurrence (Table 2), and 8 papers investigating the association between BPE and breast cancer risk (Table 3).

### 3.1. BPE Classification

Borkowski et al. used a transfer learning approach and trained a deep 2D CNN for standardized and automatic BPE classification [4]. This was the first study to attempt to qualitatively classify BPE by machine learning using whole images, similar to a human reader. Their retrospective study included 11,769 single MRI first post-subtraction images obtained at 3 T from 149 patients. A hierarchical approach implemented transfer learning with the first computational model detecting slices imaging breast tissue and the second computational model performing BPE classification. Two board-certified radiologists annotated the data. The consensus of 2 radiologists served as reference/ground truth for BPE classification. For the BPE model, training, validation, and testing subsets of 87, 25, and 12 patients were randomly selected. For the testing group, a subset of 100 images (25 for each BPE category) were utilized for the evaluation. The images were annotated by 2 radiologists with over 5 years of breast imaging experience. Accuracies of 98, 96, and 97% for training, validation, and testing, respectively, were achieved for classifying slices demonstrating breast tissue. Mean accuracies of 74, 75, and 75% for training, validation, and real-world dataset, respectively, were achieved for BPE classification of 4 classes. Inter-reader reliability for radiologist 1 was 0.780, for radiologist 2 was 0.679, and for the 2DCNN model was 0.815. The kappa coefficient for the agreement between the 2 experts and each expert with the model was 0.793 ± 0.15, 0.804 ± 0.14, and 0.768 ± 0.16, respectively. The overall accuracy of the breast detection model was 96%. The BPE model’s overall accuracy was 75%. The accuracy of reader 1 and reader 2 were 69% and 52%, respectively. The reliability of both readers differed for different BPE classes, ranging from 0.47 to 0.71 for the first and 0.24 to 0.49 for the second reader. The reliability of the second reader was lower than that of the first one except for BPE class of minimal enhancement. The reliability of the model exceeded both readers except for moderate enhancement. The main limitation of this study was the small sample size and single institution.

Eskreis-Winkler et al. developed two deep learning AI CNN models based on VGG-19 architecture for automated BPE classification (Slab AI and MIP AI) and using standard-of-care radiology report BPE designations and three-reader averaged consensus of BPE scores as ground truth [5]. The study included 5224 breast MRI, divided into training, testing and validation. On radiology reports, 1286 exams were categorized as high BPE (i.e., marked or moderate) and 3938 as low BPE (i.e., mild or minimal). Slab AI and MIP AI were tested, and cross validation was performed to evaluate performance. They found that the Slab AI model significantly outperformed the MIP AI model across the full test set (area under the curve of 0.84 vs. 0.79) using the radiology report reference standard. Using a three-reader consensus BPE labels reference standard, the Slab AI model significantly outperformed radiology report BPE labels (AUC of 0.96 versus 0.83) for BI-RADS 1. The AI model was significantly more likely to assign “high BPE” to suspicious breast MRIs and significantly less likely to assign “high BPE” to negative breast MRIs compared to the radiologist. Additionally, the AI tool can autopopulate the BPE result into the report, providing increased accuracy while reducing radiologist workload. The strengths of the study are that it included a large sample size with cross validation and consensus from three experts as ground truth. They concluded that fully automated BPE assessments for breast MRI improve accuracy over BPE assessments from radiology reports. 

Ha et al. developed a fully automated CNN method for quantification of breast MRI FGT and BPE [6]. They manually segmented and calculated the amount of FGT and BPE to establish ground truth parameters. Then, a novel 3D CNN modified from the standard 2D U-Net architecture was developed and implemented for voxel-wise prediction of whole breast and FGT margins. Cases were separated into training (80%) and test sets (20%). Fivefold cross-validation was performed. In the test set, the fully automated CNN method for quantifying the amount of FGT yielded an accuracy of 0.813 (cross-validation Dice score coefficient) and a Pearson correlation of 0.975. For quantifying the amount of BPE, the CNN method yielded an accuracy of 0.829 and a Pearson correlation of 0.955. Their CNN network was able to quantify FGT and BPE within an average of 0.42 s per MRI case.

Nam et al. developed a fully automated machine-learning algorithm for whole breast FGT segmentation and BPE classification [7]. The study consisted of 594 patients assigned to the development set, and 200 patients to the test set. Automated segmentation was performed with 3D V-NET CNN. Manual segmentation of the contralateral breast was performed for the whole breast and FGT regions. BPE was acquired by thresholding using the subtraction of the pre- and postcontrast T_1_-weighted images and the segmented FGT mask. 3 classification models were trained and tested, including conventional 4-way classification of BPE, 3-way classification of BPE (minimal versus mild/moderate/marked), and 2-way classification (minimal/mild versus moderate/marked). Two radiologists independently assessed the categories of FGT and BPE as ground truth. A deep-learning-based algorithm was designed to segment and measure the volume of whole breast and FGT and classify the grade of BPE. Dice similarity coefficients (DSC) and Spearman correlation analysis were used for evaluation. The mean DSC for manual and deep-learning segmentations was 0.85 ± 0.11. The correlation coefficient was 0.93 for FGT volume from manual and deep-learning derived segmentation methods. Overall accuracy of manual segmentation and deep-learning segmentation were comparable for BPE classification, at 66% and 67%, respectively. Binary categorization of BPE grade (minimal/mild vs. moderate/marked) increased overall accuracy to 91.5% and 90.5% for manual and deep-learning segmentation, respectively, with 0.93 AUC for both. Segmentation and classification computation time including pre- and post-processing data were under 2 min per exam. This deep-learning-based algorithm demonstrated similar accuracy to radiologists. Limitations include the single-center nature of the study and the use of radiologists’ subjective assessments as ground truth.

### 3.2. BPE for Predicting Recurrence

Arefan et al. demonstrated the use of a quantitative BPE scoring method to predict risk of breast cancer recurrence in women with breast cancer [8]. The study is a single-institution retrospective review of women diagnosed with unifocal estrogen receptor positive and node negative breast cancer between January 2007 and January 2012, with a follow-up period of 10 years. The patient population includes women who were diagnosed with invasive breast cancer including ductal, lobular, and mixed pathologies. In total, 127 women were included in the study group. Additionally, 60 women were selected as an internal test set using similar selection criteria. MRI was obtained on a 1.5 Tesla scanner using similar protocol for all patients. FGT was segmented, and BPE quantification was performed using computer models for both the contralateral whole breast and the ipsilateral breast with the exclusion of pathologic enhancement. Five BPE quantitative measures for each breast were calculated. Additionally, tumor radiomic data were extracted to further characterize the mass. Subsequently, univariable logistic regression was used to examine the association of BPE with Oncotype DX recurrence score binarized into high-risk (recurrence score > 25) and low- or intermediate-risk (recurrence score ≤ 25) categories. The study determined that of the 127 women in the study group, there was a 15.7% cancer recurrence rate (20 women). It was found that quantified BPE measurements in both breasts were associated with increased odds of a high-risk Oncotype DX recurrence score, with the highest correlation noted when calculating BPE relative to breast volume in both the ipsilateral and contralateral breast (odds ratio range, 1.27–1.66 [95% CI: 1.02, 2.56]; *p* < 0.001 to *p* = 0.04). Additionally, when combined with tumor radiomics, quantitative BPE scores helped distinguish patients with a high-risk Oncotype DX recurrence score from those with a low- or intermediate-risk score, with an area under the receiver operating characteristic curve of 0.94 in the development set and 0.79 in the test set, demonstrating the use of BPE calculation as an adjunct method of stratifying risk. This study has certain unique features, including quantification and comparison of BPE in both the breast contralateral to and ipsilateral to the known cancer, which in other studies is not examined as thoroughly. Additionally, the study demonstrates the utility of combining BPE and tumor radiomics to stratify risk, which differs from other studies that examine the relationship of BPE to tumor occurrence alone. However, the study is limited by its small sample size and retrospective nature and limited to a patient population of ER and PR positive breast cancer only, thus is not fully generalizable. Overall, the study was able to demonstrate the utility of quantitative BPE calculation in both contralateral and ipsilateral breast for determining risk of breast cancer recurrence. The study was also able to demonstrate the combined use of calculated BPE measurement and tumor radiomics for recurrence risk.

Moliere et al. retrospectively examined 102 patients with biopsy-proven unilateral invasive breast cancer treated with NAC and surgery who had pre- and post-NAC MRI [9]. BPE visually and quantitatively was assessed to predict recurrence post-NAC. Imaging at 1.5 T included 3D fat suppressed T1 pre and 5 post contrast DCE, 1st post-contrast (45 s). A total of 2 readers with 3 years of breast MRI experience, blinded to pathology data, assessed FGT and BPE on subtracted enhanced images. Disagreement was then resolved by consensus. A 2nd eval 6 months later was performed by reader 1. Inter and intra-reader variability was assessed. A semi-automated segmentation method for the unaffected breast for FGT on the T1 pre-contrast non-fat sat included supervised delineation of chest wall and segmentation by thresholding with fuzzy C-means. FGT was quantified as FGT volume divided by the volume of the entire breast. The volume of BPE is then calculated as the sum of voxels (where each voxel equals intensity in voxel on subtraction or I post minus I pre, which is then divided by intensity in the voxel pre-contrast or I pre) for all voxels ≥ 20%. There was a strong positive correlation between quantitative BPE and risk of recurrence on post NAC MRIs, but not on pre-NAC MRIs. Quantitative and visual assessment had a moderate correlation. Quantitative BPE correlated to disease-free survival (DFS) independently of PCR.

### 3.3. BPE for Predicting Risk

Hu et al. evaluated the association of breast cancer with BPE intensity (BPE_I_), BPE volume (BPE_V_), and the amount of FGT using an automatic quantitative assessment method in breast MRI [10]. The fully automated method consisted of a 3-step process, consisting of whole breast segmentation, FGT segmentation, and enhanced FGT segmentation. Subsequent calculations were performed to obtain FGT, BPE_I_, and BPE_V_. The volume ratio of segmented FGT and breast tissues generated FGT. BPE_V_ was derived from the volume ratio of enhanced to unenhanced FGT and BPE_I_ was derived from the intensity ratio of enhanced FGT. This retrospective study included 132 healthy women (control group), 132 women with benign breast lesions (benign group), and 132 women with breast cancer (cancer group) matched by age and menopausal status. For women with a benign lesion or a cancerous lesion, the contralateral breast was evaluated to avoid lesion-associated effects. Compared with the control groups, the cancer group showed a significant difference in BPE_V_ with a maximum AUC of 0.715 and 0.684 for patients in premenopausal and postmenopausal subgroups, respectively. A significant difference in BPE_V_ was demonstrated in the cancer cohort with a maximum AUC of 0.622 and 0.633 for premenopausal and postmenopausal subgroups, respectively, when compared with the benign group. FGT showed no significant difference when the breast cancer group was compared with the healthy control and benign lesion group, respectively. Compared with the control groups, BPE_I_ showed a slight difference in the cancer group. Compared with the benign group, no significant difference was seen in the cancer cohort. The novelty of the study includes automated assessment of BPE, the use of BPE_V_, and accounting for menopausal status. They concluded that increased BPE_V_ correlated with a high risk of breast cancer while FGT did not. A limitation of the study was its small sample size.

Lam et al. retrospectively evaluated a matched case–control cohort of 46 high-risk, 23 cancer, and 23 control patients [11]. A single slice from the DCE acquisition at or near the level of the nipple was selected as representative by an experienced radiologist blinded to outcome. If one breast had suspicious findings, then the contralateral breast was used. BPE maps were performed based on percent FGT enhancement in each voxel. Qualitative BI-RAD BPE were obtained as per report. Semiautomated segmentation of FGT was performed. Percent enhancement for each voxel in FGT was calculated and BPE maps were generated at varying percent-enhancement thresholds at 10% threshold increments, from 10% to 70%. BPE maps only included voxels equal to or exceeding the threshold. A total of 10 quantitative parameters were calculated for each BPE map. BPE area ratio had the highest AUC (0.76) among the BPE area parameters. 25th percentile BPE intensity had the highest AUC among BPE intensity parameters (0.77) for predicting breast cancer risk. BPE integrated intensity combined area and mean BPE, with an AUC of 0.78. BPE area, BPE to FGT ratio, and BPE intensity were associated with breast cancer, while FGT area and whole breast area were not. Quantitative BPE parameters yielded higher AUC compared to qualitative BPE, though this was not statistically significant. Limitations of this study include small sample size and that only one slice selected by an experienced radiologist was used for quantitative BPE analysis.

Niell et al. evaluated a population of high-risk patients using a semiautomated 3D segmentation algorithm to calculate quantitative BPE [12]. A total of 95 high risk women without personal history of breast cancer, of which 19 developed breast cancer, had MRI between 2010 and 2016. The 19 patients comprised the study group and age-matched controls of 76 patients were then curated, of which 62 control patients were selected who were free of breast cancer within a two-year follow-up period. They found that patients who developed breast cancer were three times more likely to have mild, moderate, or marked BPE, based on the radiologist’s assigned category. A maximum AUC of 0.62 was achieved using BPE threshold greater than minimal. This is in keeping with similar studies, as shown in a meta-analysis of seven studies by Thompson et al., where high-risk women with mild, moderate, or marked BPE demonstrated twice the risk compared to minimal BPE [13]. They also performed quantitative BPE analysis which showed that it outperformed radiologist classification, with AUC values of 0.85 and 0.84 using the first gadolinium-enhanced phase BPE at 30% and 40% enhancement ratio thresholds, respectively. The percent enhancement ratio was determined by averaging the voxels of enhancement, estimating the percentage of breast tissue that enhances above the threshold value relative to the total breast volume (BPE%). This was conducted in 10% increments to ascertain the ideal threshold for percent enhancement. They segmented FGT from chest wall/pectoralis, skin, and fat on pre- and post-contrast images to quantify FGT and BPE. The automated process was reviewed and refined by 2 scientists and then reviewed by a breast radiologist. No difference was seen in FGT category, median total breast volume, FGT volume, and FGT % between patients who developed breast cancer and those who did not. Similarly, volumetric BPE did not show a significant disparity between patients who developed breast cancer and controls, in univariate analysis. However, feature pairs that included volume and intensity of BPE showed greater sensitivity, PPV, and Youden index compared to univariate BPE%. Limitations of this study include sample size, semiautomated as opposed to fully automated segmentation, and inability to determine reproducibility and variability over time, as only the index MRI was utilized in their data. Their results were similar to those of Lam et al., who reported AUC up to 0.78 using 10 to 40% threshold on the first post-contrast images using a 2D quantitative method. 

Saha et al. examined a cohort of 133 high-risk women in a retrospective study spanning 9 years (2004–2013, 1039 high-risk patients), where 46 developed cancer over a 2 year follow-up period and 87 served as controls [14]. A total of 1.5 or 3 Tesla pre-contrast axial fat saturated and non-fat saturated and post-contrast axial fat-saturated sequences were used for data pre-processing and feature extraction. Automated segmentation of FGT was obtained with a fuzzy-C-Means clustering method. Quantitative BPE was derived from 2-D and 3-D images. A team of 5 breast radiologists reviewed exams for BPE independently with BI-RADS 5th ed. AUC was calculated using leave-one-out cross-validation. Computer models based on automated extraction features demonstrated higher AUCs than human readers. Machine learning model 1 (BPE features based on the FGT segmentation on the fat saturated sequence) demonstrated AUC of 0.52 to 0.73 and machine learning model 2 (BPE features based on the FGT mask on the non-fat saturated sequence) demonstrated AUC of 0.60 to 0.79. Mean and median reader AUCs were 0.49 to 0.70 and 0.51 to 0.69, respectively. Two thresholds were tested: minimal versus mild/moderate/marked and minimal/mild versus moderate/marked. Automatic features outperformed subjective readings for both thresholds for breast cancer prediction. Features derived from FGT mask obtained from T1 non-fat saturated pre-contrast images performed better than from FGT mask obtained from 1st post-contrast DCE. Limitations include small sample size, the retrospective nature of the study, and the reliance on a single institution.

Vreemann et al. performed a retrospective study that included negative baseline MRI scans of high-risk patients screened for breast cancer at 1.5 or 3 T MRI during the period 1–1-03 to 1–1-14 [15]. Transverse or coronal T1 GRE pre- and post-contrast images were obtained. Deep learning was implemented to quantitatively measure FGT using pre-contrast T1 GRE DCE images and BPE relative enhancement values using pre- and 1st post-contrast motion-corrected DCE acquisitions. Segmented FGT volume divided by total breast volume constituted the fraction of FGT. The fraction of BPE relative to the volume of FGT was calculated using a relative enhancement value of over 10% for any given voxel as constituting BPE. Both breasts were averaged for final BPE and FGT assessment. The same analysis was repeated using relative enhancement cut-off values of 20, 30, 40, and 50%. During the follow-up period, 60 cancers were diagnosed. Logistic regression using forward selection determined whether there were any relationships between FGT, BPE, cancer detection, false-positive recall, and false-positive biopsy. For recalls that did not lead to biopsy, at least 1 year of clinical follow-up was established as ground truth for benignity. This study found that BPE and FGT were not associated with overall breast cancer development in their high-risk population; however, it was associated with false positive recalls and false positive biopsies at baseline, which disappeared with subsequent screening rounds. Subgroup analysis of patients who developed breast cancer within 2 years of their baseline exam (17 cancers and 1499 baseline MRIs), showed that only FGT was associated with short term risk (within 2 years). BRCA mutation carriers had lower FGT and BPE and lower age at baseline scan. Similarly to this, 2 earlier studies found that mammographic increased breast density or increased FGT on MRI in BRCA positive women was not predictive [2,3]. This is in contrast to a study by Holm et al. showing that increased density in average-risk women does impact breast cancer risk [16]. Mitchell et al. reported that increased breast density in BRCA-positive patients conferred increased risk of breast cancer, similar to the relative risk in the general population [17]. Limitations include the retrospective nature of study, the use of only baseline exams which therefore does not consider alterations in BPE on subsequent exams, and potentially selection bias in only selecting exams that were negative at baseline, while including false positive exams. 

Wang et al. aimed to use deep learning to evaluate possible associations between the quantitative properties of breast parenchyma on baseline MRI scans and breast cancer occurrence among women with extremely dense breasts [18]. The study is a secondary analysis of data obtained from the DENSE trial, in which 4553 women ages 50–75 with BI-RADS category D breast density without abnormality on mammography were followed for 6 years to assess for the development of interval breast cancer. The study was performed at multiple institutions in the Netherlands between December 2011 and January 2016. Image analysis was performed on baseline MRI to segment FGT using an nnU-Net technique. Spatiotemporal characteristics of FGT were classified into 15 quantitative features, and each image-processed MRI was then analyzed by neural network to classify the FGT according to MRI feature. The 15 characteristics were then grouped into 5 principal components, and multivariable Cox proportional hazards regression statistical analysis was performed to correlate associations between each principal component and breast cancer occurrence. The study determined that there was a statistically significant association between the volume of enhancing parenchyma on baseline MRI (PC1) and breast cancer occurrence (hazard ratio [HR], 1.09; 95% CI: 1.01, 1.18; *p* = 0.02). Additionally, when stratified into low, intermediate, and high volume of enhancing parenchyma, there was nearly double the occurrence of breast cancer in the high tertile than in the low tertile (HR, 2.09; 95% CI: 1.25, 3.61; *p* = 0.005). Other imaging characteristics, such as early contrast uptake of parenchyma, shape of parenchyma, late contrast uptake of parenchyma, and breast density on MRI, were not significantly associated with breast cancer occurrence. Major strengths of this study are the prospective nature of the study design, and the large sample size. The study, however, only examines women with extremely dense breasts and is not generalizable to a larger population. Additionally, a follow up time of 6 years limits the evaluation of late breast cancer development, and no racial or ethnic data were obtained. The study concluded that quantitative parenchymal features on baseline dynamic contrast-enhanced MRI scans are independent predictors of breast cancer occurrence in women with extremely dense breasts.

Watt et al. aimed to use a fully automated quantitative measure of background parenchymal enhancement (BPE) and examine its association with odds of breast cancer development in patients undergoing breast MRI [19]. The study is a prospective multicenter study based in the USA, with a population of patients who had received a breast MRI between November 2010 and July 2017. The study population included both patients undergoing high-risk screening MRI and patients undergoing diagnostic breast MRI as part of workup. Of the 1476 patients in this group, 536 had a subsequent breast cancer occurrence, and 940 cases were included in the control group. MRI was obtained using standard protocol at each of the clinical sites. Subsequently, the MRI images were analyzed using a fully automated computational method as detailed by Wei et al. 2020, with scores generated for BPE extent and BPE intensity. Calculated results were compared with analysis from a single board-certified radiologist blinded to case–control status and clinical data. With multivariable logistic regression, statistical analysis was performed between breast cancer occurrence and tertiles of BPE extent, BPE intensity, FGT volume, and fat volume. This study determined that calculated BPE extent, defined as the proportion of FGT voxels with enhancement of 20% or more, was positively associated with radiologist-determined BI-RADS BPE (rs = 0.54; *p* < 0.001). In addition, participants with high calculated BPE extent had 74% increased odds of breast cancer (odds ratio, 1.74; 95% CI: 1.23, 2.46]) relative to participants with low BPE extent. This study was able to demonstrate the concordance of calculated BPE extent with radiologist-determined BI-RADS BPE, lending credence to the use of automated BPE calculation methods. The study is limited, however, by the disproportionate number of participants in the control group undergoing MRI for high-risk screening, thus having a greater burden of known breast cancer risk factors than the case participants did. Overall, this study demonstrates the non-inferiority of calculated BPE scores for clinical use and demonstrates the increased odds of breast cancer occurrence in those in the study population with high BPE extent.

Zhang et al. studied 80 women with early-stage invasive breast cancer who underwent MRI, of whom 46 were low risk and 34 were intermediate or high-risk based on Oncotype Dx [20]. Mean age was 51.1. All had MRI on a 1.5 or 3 T magnet with pre and 3 post contrast phase acquisitions. Automatically extracted FGT from contralateral breast with K-Means clustering algorithm on the pre-contrast T1 weighted images was performed. Risk stratification by Oncotype Dx Recurrence Score to determine if low risk, which requires no chemotherapy, showed that 46 patients or 58% were low risk and 34 or 42% were intermediate or high-risk. Overall BPE score showed no significant difference (*p* = 0.642). Intermediate/high-risk patients were more likely HER2+ (*p* = 0.029), have higher nuclear grade (*p* = 0.030), and less likely to be on hormonal treatment (*p* = 0.004) relative to the low-risk group. Initial enhancement = IE (percentage increase in signal from pre-contrast to 1st post with respect to pre-contrast), overall enhancement = OE (percentage increase from pre-contrast to last post-contrast with respect to the pre-contrast), and late enhancement = LE (percentage increase from 1st pre-contrast to last post-contrast with respect to first post-contrast) were calculated. Pre-contrast T1 was used for baseline. Pixels with negative signal post-contrast or showing over 300% enhancement were removed and deemed not physiologic. Correlation of BPE with continuous OncotypeDx score for median initial/overall/late enhancement yielded *p*-values of 0.07, 0.05, and 0.13, with AUC *p*-value of 0.06. BPE in contralateral breast correlated with ODxRS. The top 10% of BPE showed even stronger correlation for IE, OE, and LE, yielding *p*-values of 0.02, 0.02, 0.22, and 0.02, respectively. When ODxRS was dichotomized into low-risk versus intermediate/high-risk, as opposed to a continuous variable, even greater association with the mean of the top 10% of pixels for BPE was demonstrated. Limitations of this study are its small sample size, retrospective nature, and focus on a single institution.

**Table 1:** Classifying BPE.

Author (Year)	Data Sources	Data Type	# of Pts	Ground Truth	Pre-Process	ML Method	Training, Validation, Testing	AUC	ACC	Spec	Sens
Borkowski (2020) [4]	University Hospital Zurich, Switzerland	1st post Subtraction	87 training 25 validation 12 testing	4 classes; 2 radiologists		VGG16	70%, 20%, 10%		75%		
Eskreis-Winkler (2023) [5]	Memorial Sloan Kettering, USA	1 pre, 3 post, 1st post subtraction FS DCE used as input	High risk 3705 patients	2 classes; hi/low Ref #1: report Ref #2: 3 reader averaged consensus	Slab and MIP k-means clustering for segmentation	VGG-19	77%, 8%, 15% (2 test sets: BR1 and BR4/5)	Ref #1: Slab AI: 0.84 MIP AI: 0.79	Ref #2: Slab: 94% Rep: 88%	Slab: 97%, MIP: 86%, Rep: 90%	Slab: 77%, MIP: 57%, Rep: 80%
Ha (2019) [6]	Columbia University	T1 pre, post, sub	137 patients	Semiautomated masks inspected by radiologist.		U-Net	80%, 20% none		BPE: 82.9%		
Nam (2021) [7]	Seoul St. Mary’s Hospital, S Korea	T2w, DCE	594 training 200 testing	2 classes; 4 classes; 2 radiologists; consensus if disagree		V-NET	75%, 25%, none	0.93 (min versus mild/mod/marked)	4-class: 67% 2 class: 91%		

2-class BPE level: minimum/mild and moderate/marked. 4-class BPE level: minimum, mild, moderate and marked.

**Table 2:** Predicting recurrence.

Author (Year)	Data Sources	Data Type	# of Pts	Ground Truth	Pre-Process	ML Method	Training, Validation, Testing	AUC	ACC	Spec	Sens
Arefan (2024) [8]	UPMC- University of Pittsburgh School of Medicine, USA	First post	127 training 60 testing unifocal inv BC ER+, node-Oncotype DX recurrence score > 10 yr F/U	Recurrence using BPE and radiomics		Automated BPE vol (BPE-v)	68%, 32%, none	0.79 high versus low/intermed risk Oncotype Dx recurrence score	NPV = 0.97		
Moliere (2019) [9]	Strasbourg University Hospital, Strasbourg, France	Pre, 5 post	102 patients treated with NAC with pre and post NAC MRIs	Post Rx BPE; 2 independent readers	SA FGT seg Threshold based on fuzzy C-means	20% threshold to calculate BPE-v		Pre BPE not predictive of PCR or recurrence		Post BPE RPR: 84%	Post BPE RCR: 93%

**Table 3:** Association of BPE and breast cancer risk.

Author (Year)	Data Sources	Data Type	# of Pts	Ground Truth	Pre-Process	ML Method	Training, Validation, Testing	AUC or H/O Ratio
Hu (2021) [10]	Fudan University Shanghai, China	Pre, 3 post	132 control, 132 benign, 132 cancer	Calculated BPEv	Fuzzy C-means clustering FGT seg			0.68 to 0.71
Lam (2019) [11]	U of Washington School of Medicine, USA	pre-, 2 post	high risk 23 cancer 23 ctrls		SA FGT seg	BPE maps of varying PE thresholds (10/20/..70%)		0.78 BPE area 0.70 (BI-RADS)
Niell (2020) [12]	Moffitt Cancer Center Research Institute, USA	Pre, 4 Post	95 high risk pts 19 cases 76 controls				80%, 20%, none	0.85 for threshold at 30% PE
Saha (2019) [14]	Duke University School of Medicine, USA	Pre, First Post	high risk 46 cases 87 ctrls	5 independent breast radiologists	MIP		Leave one out cross-validation	0.70
Vreemann (2019) [15]	Radboud University Medical Center, Netherlands	Pre, 5 post; 1st post used for BPE	high risk 60 cancer 1473 controls	biopsy-proven cancer or negative on clinical exam	U-net (DL) for FGT segmentation	BPE at cutoffs of 20, 30, 40, 50% relative enh values		BPE not predictive for breast cancer in high-risk pts.
Wang (2023) [18]	Multicenter, DENSE clinical trial, Netherlands	Pre, 4 or 5 post	122 cancer, 4431 ctrls	Breast radiologists	nnUNet for FGT segmentation	Quantitative features including enhancement characteristics		BPE HR = 1.09 [95% CI: 1.01, 1.18]
Watt (2023) [19]	Multicenter, IMAGINE clinical trial, USA	Pre, Post, Sub 1st post	536 cancer 940 ctrls	single board-certified radiologist		Automated method; proportion of voxels enhancing > 20%		High BPE OR = 1.74 [95% CI: 1.23, 2.46]
Zhang (2021) [20]	Memorial Sloan Kettering, USA	Pre, 3 post	46 low risk 34 interm/high risk by Oncotype Dx. Early-stage invasive BC	Manually by radiologist. Association of BPE with Oncotype DX	k-means clustering algorithm to extract FGT			

Footnote: Only two studies were prospective (Wang et al. and Watt et al.). The rest were retrospective.

## 4. Discussion

### 4.1. Types of Ground Truths

Ground truth for BPE used in classification is typically scored by radiologists as minimal, mild, moderate, and marked. BPE can also be calculated based on different thresholds of percentages of post-contrast signal enhancement. For ML to perform BPE estimation, it needs to learn breast tissue versus non-breast tissue and FGT versus fat tissue. ML was often used to automatically segment FGT, for which manual contouring or automated segmentation based on fat-water MRI is used as ground truth. Quality of ground truth is critically important. 

### 4.2. Data Inputs and Sample Sizes

Input data typically included pre-contrast, first post-contrast, and multiple post-contrast. Multiple post-contrast MRI dynamics can be included to improve accuracy. It is not clear whether using all post contrast MRI dynamics would help classification, as there are no vigorous studies directly addressing this question. It is conceivable each subsequent post-contrast MRI dynamic could add additional information, such as improving SNR, but contrast-to-noise ratio decreases with subsequent dynamics compared to using only the first post-contrast. In addition, potential motion between dynamics could have negative effects. It is possible to improve performance if different post-contrast MRI dynamics can be weighted differentially. 

Sample sizes mostly were in the low hundreds, with two studies having ~4000 subjects, which is generally considered to be small. 

### 4.3. Types of ML Methods

Several of the studies implemented CNN models using systems such as U-net, VNET, VGG-16, and VGG-19. Generally, the highest performance was demonstrated by DL-driven models, demonstrating better accuracy than human readers [5]. There could, however, be publication bias in which papers demonstrating favorable performance are more likely to be published. Additional and independent testing on independent datasets is needed. DL models may potentially be designed to automatically provide accurate BPE information as well as incorporate and input other imaging and clinical data to help guide in decision-making, thus superseding a mere quantitative analysis.

### 4.4. Classification

Binary classification of BPE gave better results compared to the conventional 4 grade system of BPE classification and different binary classifications may produce slightly different results. Nam et al. achieved an AUC as high as 0.93 and accuracy of 91% using a deep learning model with automated whole breast segmentation and BPE classification when a binary classification of BPE was used (min versus mild/mod/severe), compared to 67% accuracy with the conventional 4 level classification system for BPE [7]. Eskreis-Winkler achieved a diagnostic accuracy of 94% with the Slab AI model using a two-class designation for BPE [5]. In their study the 2 classes were high (moderate/marked) versus low (minimal/mild). Decreasing the number of classification levels from a quaternary to a binary system appears to improve accuracy with possibly better results when min/mild and moderate/severe are utilized ostensibly as surrogates for low and high BPE. Demarcating cases as low (minimal/mild) versus high (moderate/marked) BPE better aligns with the prevailing thought that a high-BPE environment is more biologically active and favorable for carcinogenesis, as opposed to a binary categorization of cases as minimal versus mild/moderate/severe, which arguably mixes biologically inactive low-BPE cases (mild) in with biologically active high-BPE cases (moderate/severe).

Fully automated quantification of FGT and BPE using DL makes its debut as a feasible technique [6]. DL was shown to outperform radiology reports of BPE designation, where consensus of 3 radiologists served as ground truth [5]. Automatic texture feature analysis was shown to outperform subjective readings for breast cancer prediction [14].

### 4.5. Predicting Recurrence

The association of BPE (based on subjective radiologist score) with cancer risk has been well reported. ML/DL can be potentially used to incorporate BPE in the prediction of breast cancer recurrence. Moliere et al. demonstrated that BPE on post-NAC MRI positively correlated with recurrence as an independent risk factor [9]. Two studies performed comparing BPE to Oncotype Dx recurrence scores both demonstrated an association of BPE with Oncotype Dx recurrence score [8,20]. Although these studies support incorporating DL/ML assessments of BPE in predicting breast cancer recurrence, the published literature to date involves relatively small cohorts. Validation on a larger scale is necessary to provide sufficient evidence for routine clinical use. 

### 4.6. Predicting Cancer Risk

Several studies performed on specifically high-risk patients demonstrated an association of BPE with increased risk [11,12,14]. It is surprising that, in a study on high-risk patients, BPE on baseline MRI was not predictive of breast cancer development in a high-risk population [15]. Unlike the other studies, they excluded cases where breast cancer was detected on baseline MRI or within 6 months, which might account for the difference in their results [15].

## 5. Challenges for DL to Achieve Routine Clinical Applications

While machine learning (ML) analysis holds promise for breast cancer evaluation, several limitations must be addressed before its integration into clinical workflows. These include data quality/diversity, interpretability, generalization, clinical validation, regulatory approval and integration, and ethical and legal considerations [21,22].

Data Quality and Diversity: Procuring extensive, diverse, and accurately labeled datasets for effective training is challenging due to variability in imaging protocols, data acquisition techniques, and labeling inconsistencies across institutions [21,22]. Collaborative efforts among institutions can help create centralized repositories of annotated datasets. Utilizing synthetic data generation and data augmentation techniques can enhance the diversity of training datasets. Additionally, establishing standardized protocols for imaging and labeling can improve consistency across datasets.

Interpretability: ML models often operate as black boxes, providing little insight into their predictions. For clinical acceptance, it is crucial to develop interpretable ML models that elucidate the features driving predictions, particularly in complex medical imaging like breast MRI. Techniques such as SHAP (SHapley Additive exPlanations) or LIME (Local Interpretable Model-agnostic Explanations) can be employed to enhance interpretability. Training models with inherently interpretable architectures, such as decision trees or attention-based models, can also provide clearer insights.

Generalization: Models trained on one dataset may not generalize well to unseen data, especially with differences in patient demographics, imaging protocols, or equipment. Employing transfer learning and domain adaptation can help models generalize better across varied datasets. Regularization techniques and robust validation frameworks should be applied to test models on diverse populations.

Clinical Validation: Promising performance in research settings does not guarantee clinical utility. Rigorous validation through prospective studies and real-world deployment is necessary to assess the impact of ML models on clinical outcomes, including diagnostic accuracy and patient management. Engaging clinicians early in the development process and conducting multi-center trials can ensure that models are validated across different clinical settings. Continuous monitoring and iterative improvements based on real-world feedback will enhance the robustness of these applications.

Regulatory Approval and Integration: ML models must comply with regulatory requirements and integrate seamlessly into existing healthcare workflows. Obtaining regulatory approval, such as FDA clearance, can be resource-intensive and time-consuming. Early engagement with regulatory bodies can clarify requirements and streamline the approval process. Developing models that are modular and easily adaptable to existing systems can facilitate smoother integration into clinical workflows.

Ethical and Legal Considerations: ML applications in healthcare raise ethical issues, including patient privacy, data security, algorithmic bias, and transparency. Implementing strict data governance policies, conducting bias audits on training datasets, and maintaining transparency in model development can mitigate ethical concerns. Educating stakeholders about responsible AI use and ensuring compliance with legal frameworks will further safeguard patient interests.

By addressing these challenges through collaborative efforts, innovative methodologies, and a focus on ethical practices, the path toward integrating ML into routine clinical applications can be significantly strengthened.

## 6. Conclusions

An automated BPE assessment will eliminate subjectivity and provide an efficient and objective metric that can be applied to risk stratification. BPE is also a biomarker for risk and prognosis in the setting of post-NAC. The ramifications of this are wide-reaching, and fully automated BPE assessment is needed to achieve its full potential. Adding BPE to clinical data such as age, family history, and genetic mutations, may improve prognosis and risk prediction. A future research direction includes the incorporation of larger datasets and additional clinical data (such as age, family history, or genetic mutations) to improve diagnostic accuracy and enable personalized medicine. 

Automated BPE assessment could enhance clinical practice by reducing subjectivity and providing a standardized metric for risk stratification. BPE aids in informed treatment decisions. 

## Abbreviations

BPE	background parenchymal enhancement
FGT	fibroglandular tissue
MRI	magnetic resonance imaging
CNN	convolutional neural network
AUC	Area under the curve
AI	Artificial intelligence
DCE	dynamic contrast enhancement
